# Tdnn-Based Engine In-Cylinder Pressure Estimation from Shaft Velocity Spectral Representation

**DOI:** 10.3390/s21062186

**Published:** 2021-03-20

**Authors:** Andrés F. Valencia-Duque, David A. Cárdenas-Peña, Andrés M. Álvarez-Meza, Álvaro A. Orozco-Gutiérrez, Héctor F. Quintero-Riaza

**Affiliations:** 1 Automatics Research Group, Engineering Faculty, Universidad Tecnológica de Pereira, Pereira PC 660001, Colombia; dcardenasp@utp.edu.co (D.A.C.-P.); aaog@utp.edu.co (Á.A.O.-G.); 2 Signal processing and Recognition Group, Universidad Nacional de Colombia sede Manizales, Manizales PC 170004, Colombia; amalvarezme@unal.edu.co; 3 Manufacturing Processes and Machine Design Research, Mechanical Engineering Faculty, Universidad Tecnológica de Pereira, Pereira PC 660001, Colombia; hquinte@utp.edu.co

**Keywords:** engine in-cylinder pressure, shaft velocity, neural networks, data prediction

## Abstract

Pressure is one of the essential variables to give information about engine condition and monitoring. Direct recording of this signal is complex and invasive, while angular velocity can be measured. Nonetheless, the challenge is to predict the cylinder pressure using the shaft kinematics accurately. In this paper, a time-delay neural network (TDNN), interpreted as a finite pulse response (FIR) filter, is proposed to estimate the in-cylinder pressure of a single-cylinder internal combustion engine (ICE) from fluctuations in shaft angular velocity. The experiments are conducted over data obtained from an ICE operating in 12 different states by changing the angular velocity and load. The TDNN’s delay is adjusted to get the highest possible correlation-based score. Our methodology can predict pressure with an R2 >0.9, avoiding complicated pre-processing steps.

## 1. Introduction

The accelerated development of internal combustion engines in recent years has enhanced the complexity of their composing elements. As a result, the machinery monitoring task gains relevance for the progressively difficult and correct engine calibration and operation [1]. Among the many variables to watch, the in-cylinder pressure of an internal combustion engine (ICE) provides the most information about the combustion process needed to optimize fuel consumption, improve machinery efficiency, and reduce pollution rates [2]. For instance, homogeneous charge compression ignition (HCCI) combustion significantly reduces emissions while maintaining a high fuel efficiency [3]. However, HCCI demands a complex control of the combustion process that the pressure measuring can alleviate [4,5]. As another application, cylinder pressure measuring revealed the influence of fuel borne additives on ternary fuel blend operated in single-cylinder diesel engines [6]. Another study analyzed the effect of fuel injection pressure on a diesel engine run with butanol-diesel blend by measuring the in-cylinder pressure signal [7]. Such a signal also allowed correlating the advancing pilot injection timing on the engine performance, combustion, and emissions under high loads with variation pilot injection timing [8]. Therefore, researchers put effort into implementing reliable pressure monitoring for accurately modeling the engine behavior [9].

Conventionally, directly measuring the pressure requires installing transducers in the combustion chamber, which is highly invasive and usually carried out under laboratory conditions. In the commercial scenario, engines lack the sensors needed due to the high cost and short useful life [10]. Therefore, several techniques estimate the in-cylinder pressure signal from non-intrusive measurements such as vibration [11,12,13,14], crankshaft position [15], and other easily measured variables [16]. Despite the vibration signals reach reasonable prediction rates, locating the accelerometers over the engine is an open research field, even being necessary to add more sensors to cope with the spatial variability [17,18,19]. The heat transfer and release rates have also been considered to be wrapper features to estimate pressure from a mathematical model [20,21]. Nonetheless, the task of inferring the heat transfer demands assumptions as treat engine gases as ideals, which cannot be true [22,23], reducing the prediction performance at varying recording conditions [24].

The crankshaft kinematics bypasses the inherent spatial uncertainty of vibration recordings relying on detailed mathematical models as the developed in [25], where the authors estimate in-cylinder pressure from shaft speed fluctuations in an ICE. Their experiments show that the faster the engine, the more complicated the crankshaft motion, and the least accurate the model. In [10] the crankshaft acceleration is used to estimate cylinder pressure accurately. However, the hand-crafted feature kinematic extraction from the crank position demands powerful machine learning tools, as the recurrent neural networks, to unravel the patterns related to the pressure signal non-linearly. In [26], the authors develop a single hidden layer feedforward neural network to predict the pressure of a spark-ignition single-cylinder engine (achieving an R2 score of 0.993) from crankshaft angle, duct pressure, and temperature signals. Similarly, the work presented in [27] developed a model to estimate the pressure in a single-cylinder HCCI engine using a multilayer neural network, operating as predictors the crank angle and the engine speed. In [28], the authors introduced a unified approach to reconstruct engine cylinder pressure using time-delay neural networks with crank kinematics or block vibration measurements. The authors in [29] estimate a four-cylinder engine pressure using a Kalman Filter-based approach from structural vibration signals. Indeed, they evaluate the coherence between cylinder pressure and vibrations records. Of note, the combustion process periodicity allows extracting relevant information from spectral analysis to develop straightforward methodologies with enhanced interpretation. For example, the spectrum of in-cylinder vibration and acoustic pressure signals provided relevant harmonics for diagnosing the combustion [30] and evaluating the working regimen [14].

In this paper, a time-delay neural networks (TDNN)-based approach is proposed for in-cylinder pressure prediction in an ICE from crankshaft angular position fluctuations. To this end, the TDNN is introduced as a Finite Impulse Response (FIR) filter perspective by taking advantage of the propagation equations of feed-forward networks. A spectral analysis through the Fourier series is used to obtain the most relevant signal harmonics according to the prediction task. Pressure estimation is carried out from the angular velocity signal directly measured from the shaft with a cheap and easily constructed encoder. The recording system acquired signals on twelve different operating states of the engine by varying loads and velocities. The TDNN performance is validated mainly using the R2 score and the Magnitude-Squared Coherence (MSC) spectral metric. For the latter, an MSC over 0.9 in the harmonics explaining 90% of the signal pressure power proves that TDNNs outperform more elaborated methodologies from state-of-the-art [10,11,12,13,14,15,16,25]. Also, the peak-based measures are computed for comparison purposes. Besides, the pressure derivative is calculated from the TDNN predictions to get insights about possible heat transfer and release rates estimation enhancement [20,21]. It is worth noting that the proposed pipeline lacks complex preprocessing stages as the TDNN performs as a data-tuned filter.

The manuscript is organized as follows: Section 2 describes the TDNN as FIR filter and the performance metrics. Section 3 shows the experimental set-up. Section 4 depicts the results and discussion. Lastly, Section 5 presents the work conclusions and future research directions.

## 2. Methods

### 2.1. Time Delay Neural Network Fundamentals

**Figure 1:**
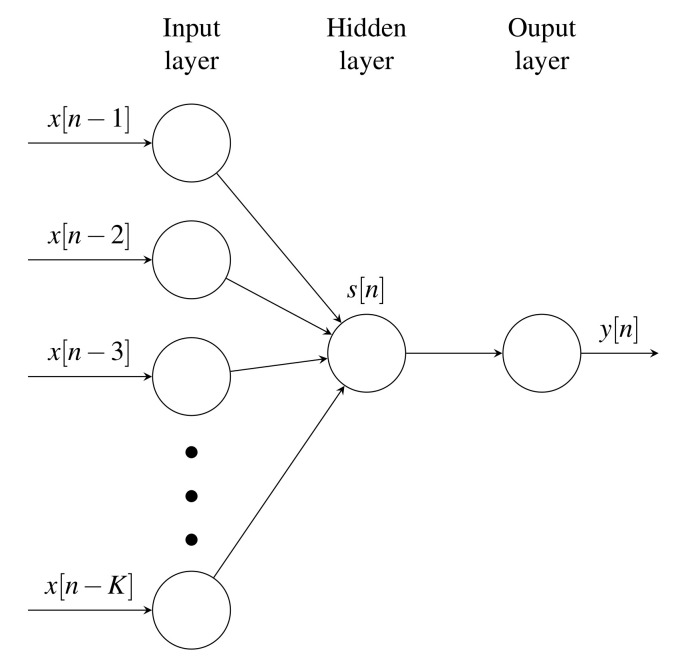
Single-layered TDNN pipeline. The network sequentially predicts the *n*-th sample in y[n] based on *K* delayed samples of x[n]. Please note that *K* directly reveals the number of units (TDNN complexity).

Artificial neural networks (ANNs) simulate biological systems that learn, store, and retrieve information, allowing them to solve classification, regression, and filtering tasks [31]. The Time delay neural network (TDNN) architecture solves time-series regression tasks by unraveling temporal patterns with an endowed input memory. TDNNs learn relationships not only among patterns but also among their sequences [32]. Figure 1 exemplifies a single-layered TDNN-based regressor from the input-output signal set ${\left\{y\left[n\right],x\left[n\right]\in R\right\}}_{n=1}^{N}$, where N∈N is the signal length. Please note that predicting the whole output signal y[n] implies the sequential application of the TDNN N−K times.

In this sense, the TDNN pipeline is mathematically described as follows:

(1a) $$\begin{array}{cc} a[n]= & \sum_{k=0}^{K}w_{k}x\left[n-k\right], \end{array}$$

(1b) $$\begin{array}{cc} s[n]= & g\left(a[n]\right), \end{array}$$

(1c) $$\begin{array}{cc} y[n]= & \nu_{1}s\left[n\right]+\nu_{0}, \end{array}$$

where n∈{1,2,⋯,N}, k∈{1,2,⋯,K}, K≤N is the maximum time delay, a[n]∈R holds the weighted sum of delayed inputs at the *n*-th instant, g(·)∈R is a non-linear activation function, and $w_{k},\nu_{1},\nu_{0}\in R$ are the network weights computed through the back propagation algorithm. Namely, the output of a hidden neuron at the *n*-th sample can be written in terms of the convolution operation:

(2a) $$\begin{array}{cc} a[n]= & \sum_{k\in Z}h\left[k\right]x\left[n-k\right]=\left(x*h\right)\left[n\right], \end{array}$$

(2b) $$\begin{array}{cc} s[n]= & g\left((x*h)[n]\right), \end{array}$$

(2c) $$\begin{array}{cc} h[k]= & \left\{\begin{array}{cc} w_{k} & :k\in \{1,2,\cdots ,K\}\\ 0 & :\mathrm{otherwise} \end{array}\right., \end{array}$$

where * stands for the convolution operator. Besides, the convolution commutativity allows computing a[n] as a linear combination of delayed weights:

(3) $$a\left[n\right]=\sum_{k\in Z}x\left[k\right]h\left[n-k\right].$$

Then, the argument of the activation function corresponds to the output of a linear time-invariant (LTI) system with the following characterizing impulse response h[n]∈R:

(4) $$h\left[n\right]=\sum_{k\in Z}w_{k}\delta \left[n-k\right]=\sum_{k\in Z}h\left[k\right]\delta \left[n-k\right]=\left(\delta *h\right)\left[n\right],$$

where δ[n]∈{0,1} corresponds to the delta Dirac function. Therefore, the single-layered TDNN non-linear maps the amplitude output of a finite impulse response (FIR) filter with coefficients learned in a supervised scheme. Since h[n] performs as an *N*-sampled impulse response FIR filter, the corresponding Fourier transform H(f)∈C, where f∈R indexes the frequency in Hz, approximates the filter transfer function [33].

In the case of periodic discrete input time series, a finite linear combination of harmonically related complex exponentials represents x[n] as:

(5) $$x\left[n\right]=\sum_{r=0}^{N-1}c_{k}e^{jrf_{0}n},$$

where $f_{0}=2\pi /N\in R^{+}$ is the fundamental frequency and $c_{r}\in C$ holds the coefficient for the *r*-th harmonic. Based on the convolution linearity property, the filter output results from gathering the convolution of its impulse response and each complex exponential:

(6) $$a\left[n\right]=\sum_{r=0}^{N-1}c_{r}h\left[n\right]*e^{jrf_{0}n}.$$

In the frequency domain, the spectral decomposition of a[n], termed A(f)∈C, becomes:

(7a) $$\begin{array}{cc} A(f)= & \sum_{r=0}^{N-1}c_{r}H\left(f\right)\delta \left(f-rf_{0}\right), \end{array}$$

(7b) $$\begin{array}{cc} A(f)= & \left\{\begin{array}{cc} llc_{k}H\left(rf_{0}\right) & :f=rf_{0}\\ 0 & :f\neq rf_{0} \end{array}\right.. \end{array}$$

Due to Equation (7a) proving that the filter output only holds spectral components at the input harmonics, the network output in Equation (1c) is rewritten as:

(8) $$y\left[n\right]=\nu_{1}g\left(\sum_{r=0}^{N-1}c_{r}H\left(\frac{2\pi r}{N}\right)e^{j\frac{2\pi rn}{N}}\right)+\nu_{0}.$$

Consequently, a TDNN fed by a periodic signal designs an FIR filter with frequency gain weighting the input harmonics to fit the target output.

### 2.2. Harmonic Prediction Performance Based on Magnitude-Squared Coherence

Given two one-dimensional periodic signals y[n] and $\tilde{y}\left[n\right]$ of period *N*, i.e., the target and TDNN-based output records, respectively; their cross-correlation function, defined in Equation (9), is also an *N*-periodic signal, where $y^{*}\left[n\right]$ denotes the complex conjugate of y[n]:

(9) $$R_{y\tilde{y}}\left[n\right]=\left(y*\tilde{y}\right)\left[n\right]≜\sum_{m\in Z}y^{*}\left[m\right]\tilde{y}\left[m+n\right].$$

Therefore, the discrete Fourier transform of $R_{y\tilde{y}}\left[n\right]$, termed the cross-power spectrum $P_{y\tilde{y}}\left[r\right]$, only holds spectral components at the fundamental frequency harmonics, i.e., $\omega_{r}=2\pi r/N$. Being provided with the cross power spectrum and the two auto power spectra $P_{yy}\left[r\right]$ and $P_{\tilde{y}\tilde{y}}\left[r\right]$, the Magnitude-Squared Coherence (MSC) is defined as the ratio between the cross power spectral density and the auto power spectrum magnitudes at the *r*-th harmonic:

(10) $$MSC\left[r\right]=\frac{{\left|P_{y\tilde{y}}\left[r\right]\right|}^{2}}{|P_{yy}\left[r\right]||P_{\tilde{y}\tilde{y}}\left[r\right]|}\in \left[0,1\right],$$

where |·| stands for the absolute function. The MSC corresponds to a zero-one bounded function, being zero when y[n] and $\tilde{y}\left[n\right]$ are uncorrelated at harmonic *r* and one if they perfectly match. Hence, MSC can be interpreted as a spectral similarity measure that quantifies the TDNN’s quality when predicting the target sequence y[n].

## 3. Experimental Setup

### 3.1. Engine Measurements, Data Acquisition, and Preprocessing

**Figure 2:**
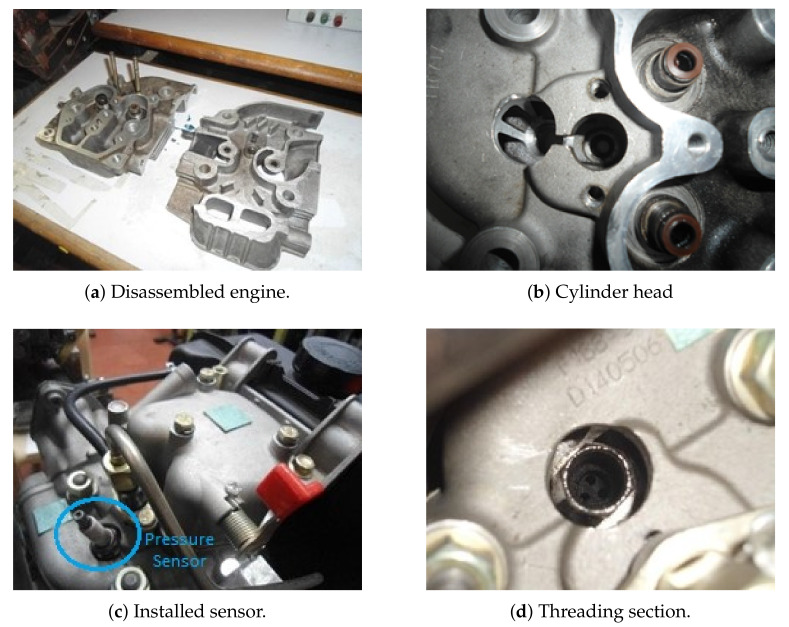
Pressure sensor implementation on a diesel-fueled CHANGFA 186 F single-cylinder engine.

**Figure 3:**
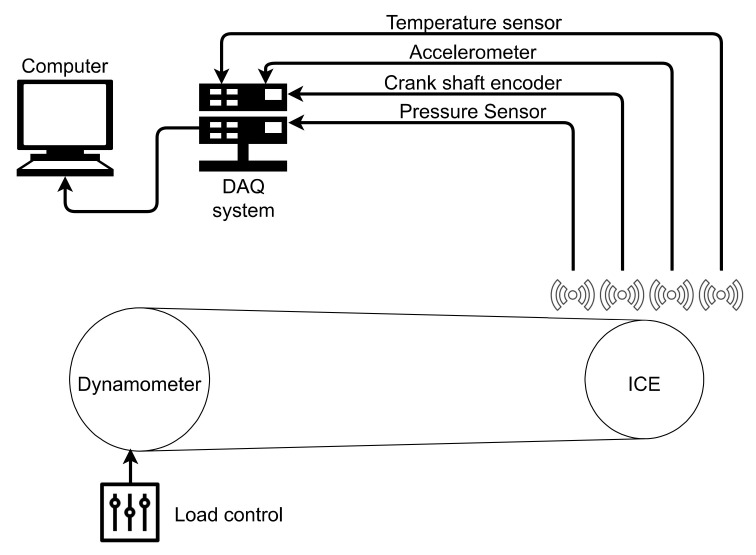
Test bench for recording signals from an internal combustion engine.

This work considers data recorded from a diesel-fueled CHANGFA 186 F single-cylinder engine, commonly used in civil engineering, energy generation, and agricultural assistance. Table 1 summarizes the CHANGFA 186 F main properties. A CFK-200 dynamometer is also used to simulate the engine load, with technical specifications shown in Table 2. Mechanical engineers installed three National Instruments data acquisition systems (DAQ) to register temperature (NI 9211), vibrations (NI 9234), pressure, speed, and angular position (NI 9222). An adaptation in the cylinder head was made for two purposes: starting the engine with provoked-ignition fuels and installing a pressure sensor in the combustion chamber. In this sense, a thread was made in the cylinder head to establish an element that would carry the spark plug (burner), because, without it, the spark plug can hit the piston. Afterward, a cylinder head identical to that of the engine was sectioned. The angle and position were determined to perform the drilling without damaging the Gas distribution system and the Diesel power system. The latter aims to guarantee a correct position and seat of the spark plug and the pressure sensor. Figure 2a shows how the cylinder head, while Figure 2b shows how the perforation was performed. Similarly, Figure 2c,d presents the installed sensor and the threading section, respectively. Then, by mounting the three DAQs over a cDAQ-9172 chassis, Matlab software synchronized engine signals. Figure 3 illustrates the component setup for the developed test bench. Furthermore, the angular position is considered for predicting the pressure due to their known kinematic relationship [14]. For the latter, a spark plug Optrand D822J6-SP sensor, installed in the engine head, measures the in-cylinder pressure y[n] at 51,200 Hz, with n∈N indexing the time instants. Table 3 introduces the pressure sensor technical specifications.

Regarding the angular position, a Hall effect sensor, attached to a 60-2 phonic sprocket wheeling with the engine shaft, generates a train of square pulses at 51,200 Hz providing information about the crankshaft angular position with a precision of 60 points per cycle (6${}^{\circ }$). Thanks to removing two out of the 60 original teeth from the sprocket, the most extended pulse marks a revolution. The raw angular velocity of $\hat{\omega }\in R^{+}$ [RPM] from the pulse train signal is computed as follows:

(11) $${\hat{\omega }}_{i}=\frac{\left(6/360)[cycles\right]}{\left(t_{i}-t_{i-1}\right)*\left(1/60\right)\left[minutes\right]}=\frac{1}{t_{i}-t_{i-1}},$$

where $t_{i}\in R^{+}$ stands for the *i*-th rising edge time instant, so that $t_{i}-t_{i-1}$ corresponds to the elapsed time for a six-degree turn. Then, a cubic interpolation-based approach [34], as presented in Equation (12), smoothly up-samples ${\hat{\omega }}_{i}$ for evening the sampling frequency of the angular velocity and the pressure signal as follows:

(12) $$x\left[n\right]=\sum_{d=0}^{3}b_{id}{\left(t_{n}-t_{i-1}\right)}^{d}\;\;\;\;\;\forall t_{i-1}\leq t_{n}<t_{i}$$

where coefficients $b_{id}$ are piece-wise computed from ${\hat{\omega }}_{i}$. Though the initial angular velocity sample rate stands for 60 Hz, the employed cubic interpolation allows coupling both the velocity and pressure dynamics to favor the prediction results. Of note, angular velocity sensors with higher sample rates can be coupled with our approach without losing the framework purpose. Here, the introduced methodology aims to probe how a straightforward and cheap scheme achieves appropriate pressure estimation results.

**Figure 4:**
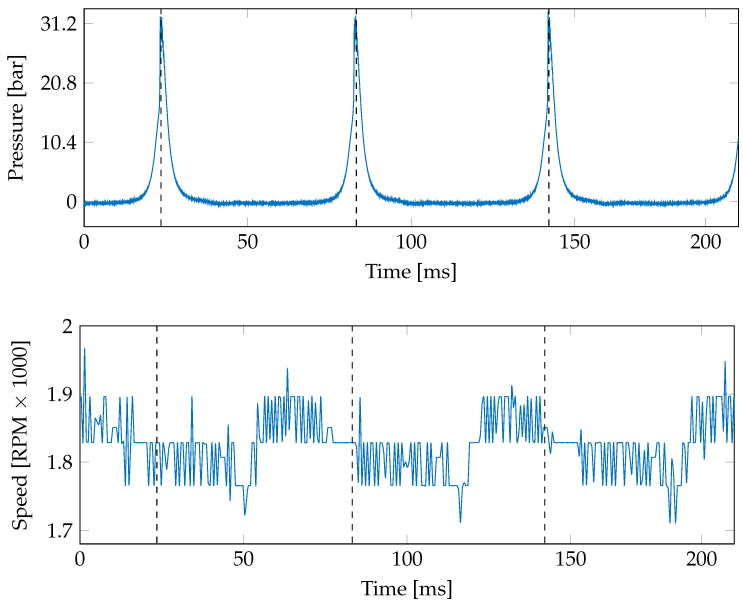
Examples of the pressure and angular velocity signals. The engine velocity and load are fixed to 1800 RPM and 3.22 BMEP, respectively. Please note that the velocity variations around 1800 RPM allow predicting the pressure signal based on our TDNN approach.

In turn, a database holding 10 second records at a sampling rate of 51.2 kHz is obtained by varying engine angular velocity and load. Three speeds (1800, 2400, and 3000 RPM) and four current induced loads in the dynamometer (0, 0.5, 1, and 1.5A) are tested (see Table 4 presented the studied loads). Figure 4 exemplifies the recorded pressure and angular velocity signals. Please note that velocity fluctuations change around the peak pressure. This fact indicates that fluctuations in angular velocity signals give information about mechanical events, for instance, intake and exhaust valve opening and closure and piston slap. Hence, the correlation between signals can be exploited to predict variables that are difficult to record in real-world environments.

### 3.2. Pressure Signal Estimation

**Figure 5:**
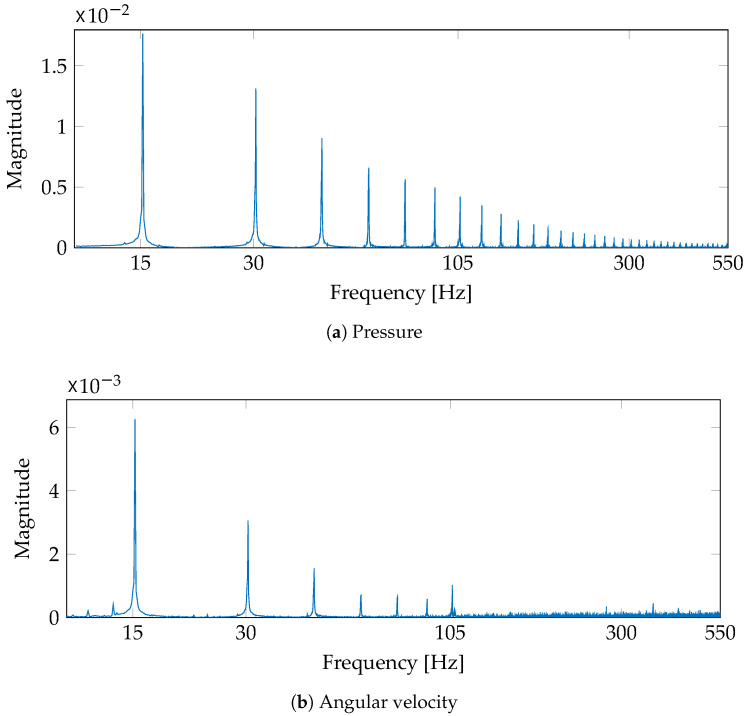
Spectrum magnitude of the pressure and angular velocity signals depicted in Figure 4 (the engine velocity and load are fixed to 1800 RPM and 3.22 BMEP, respectively). The amplitude is normalized concerning the first harmonic power. The horizontal axis is on a logarithmic scale.

The considered pressure prediction from shaft angular velocity fluctuations holds three stages: Firstly, signals are down-sampled to a fifth of the main sampling frequency. All the relevant spectral components lay under 550 Hz (see Figure 5). Secondly, the number of time delays (number of units) from the single-layered TDNN is tuned in a cross-validated scheme that searches for the most massive R2 within a grid from 100 to 2000, every 100 delays, and from 2500 to 5000, every 500 delays, yielding 26 different network architectures. Each architecture is trained five times to select the best performing trial. The cross-validation scheme divides each 10-second recording into a training (second 1 to 6) and a testing (second 6 to 10) segment, being the former split into 90% for learning the parameters and 10% for validation purposes.

## 4. Results and Discussion

**Figure 6:**
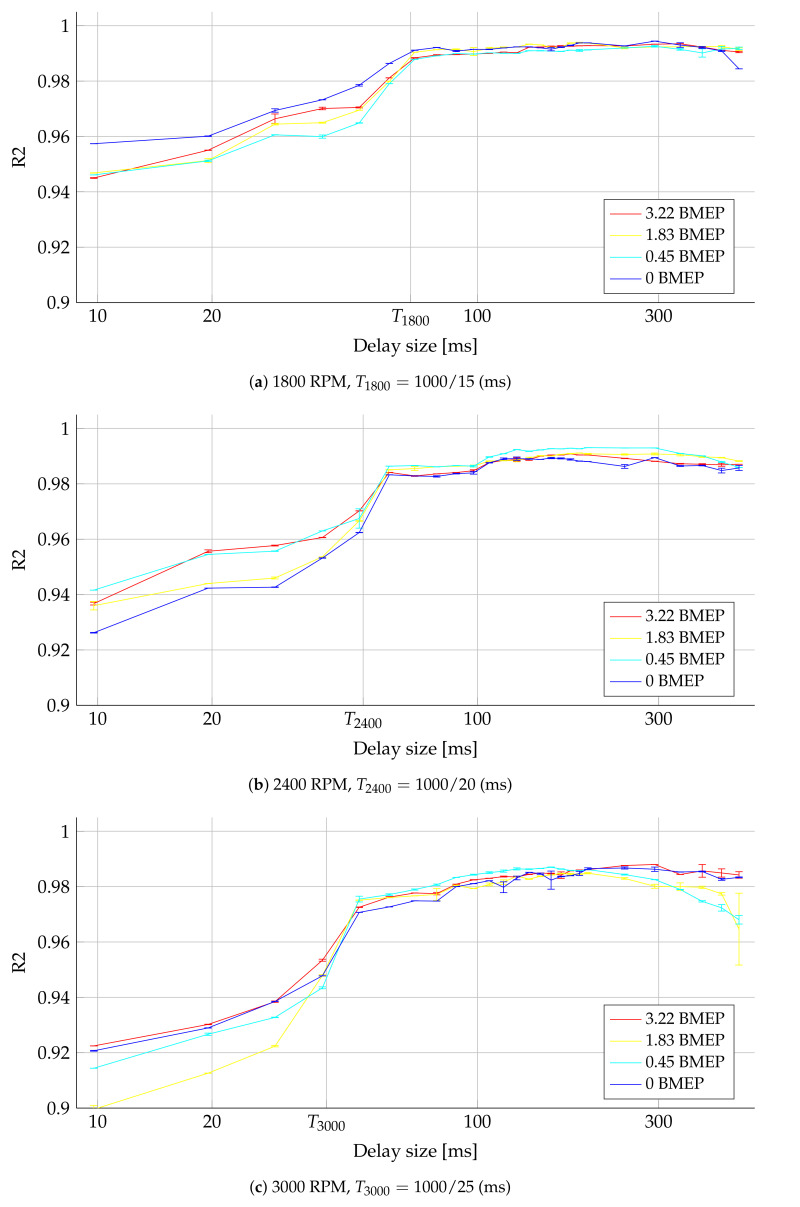
R2-based quality assessment results for tunning the TDNN’s time-delay (network complexity). The testing set results are depicted (average and standard deviation as error bars) by varying: the time delay (TDNN’s number of units *K*), the speed (in rpm), and the load (applied current to the dynamometer in amperes). The horizontal axis is on a logarithmic scale. The fundamental period is highlighted as $T_{RPM}$.

Figure 6 reports the R2-based assessment. The time delay (TDNN complexity represented by the number of units *K*), the engine velocity (in rpm), and the load (as the current applied to the dynamometer) are studied. The $T_{RPM}$ value marks the time delay matching the fundamental period as a reference value to analyze the prediction assessment. As seen, R2 values above 0.9 are obtained regardless of the fixed time delay, speed, and load. Hence, our TDNN-based strategy is robust against different engine configurations, which validates the proposed scheme’s capability for cylinder pressure prediction. Namely the introduced TDNN can be appropriately adapted regarding the required time-delay (network complexity) to deal with the minimal and maximal engine operation conditions (as exposed in Table 4). Overall, our strategy can be adapted to the predictor behavior regarding the required network representation space (time delay). Notably, the performance increases over 0.95 after the fundamental period reference as time delay ($T_{RPM}$). Indeed, the R2 performance for time-delays between $T_{RPM}$ and 300 ms remains steady. Beyond such a limit, the performance presents a slight decrease, possibly due to the TDNN’s overfitting. Nonetheless, predictions above 0.9 are always attained. Therefore, a suitable time delay is fixed at 100ms as it provides the best trade-off between performance and model complexity.

**Figure 7:**
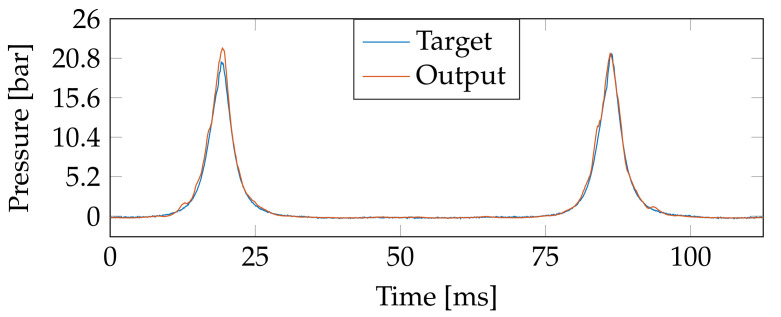
Time courses of the TDNN prediction for an unseen test subset. The target and output pressure prediction are presented for the following set-up: engine running at 2400 RPM with 0.45 BMEP load. The time delay is fixed as 100 ms.

**Figure 8:**
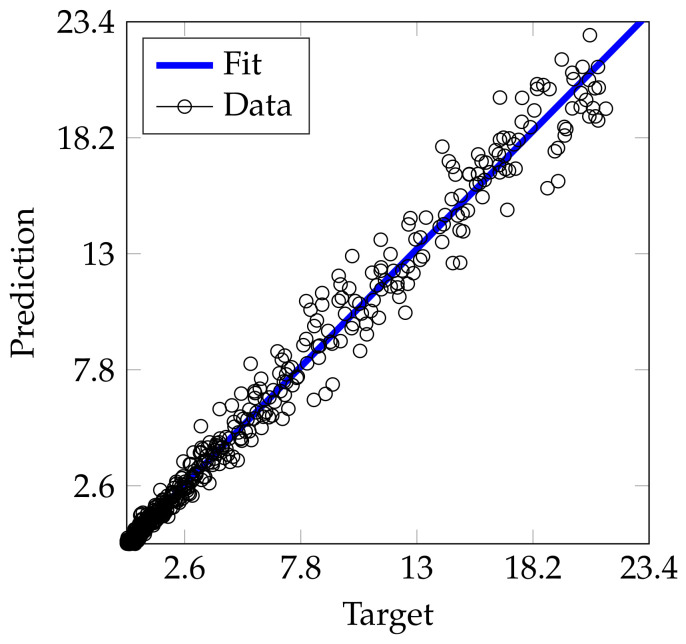
Regression plot (target vs. TDNN-based prediction) for the test subset presented in Figure 7. The *o* represents a given sample. Blue line: mean square error-based fitting (target vs. prediction along with the studied samples) holding a R2=0.994.

Now, as an illustrative example, Figure 7 plots the target (blue) and output prediction (orange) for a test pressure sample with the engine running at 2400 RPM with 0.5 A load in the dynamometer (time delay is fixed as 100 ms). As seen, the TDNN prediction properly follows the pressure trend. The performance slightly reduces near the pressure peaks due to the higher-order harmonics existing in this plot section. However, our approach achieves a decent estimation of the target signal. Besides, the R2 provides high-performance rates despite such errors. Notably, the number of high-pressure time instants is considerably smaller than the low-pressure ones where the low frequencies are dominant. The regression plot in Figure 8 (target vs. TDNN-based predictions of the samples studied in Figure 7) proves that most of the data is located at a low pressure where the TDNN easier predicts the output due to the slow dynamics. By contrast, high-pressures present larger prediction errors related to the drastic changes near the signal peaks but preserving a reasonable estimation in general (R2=0.994).

**Figure 9:**
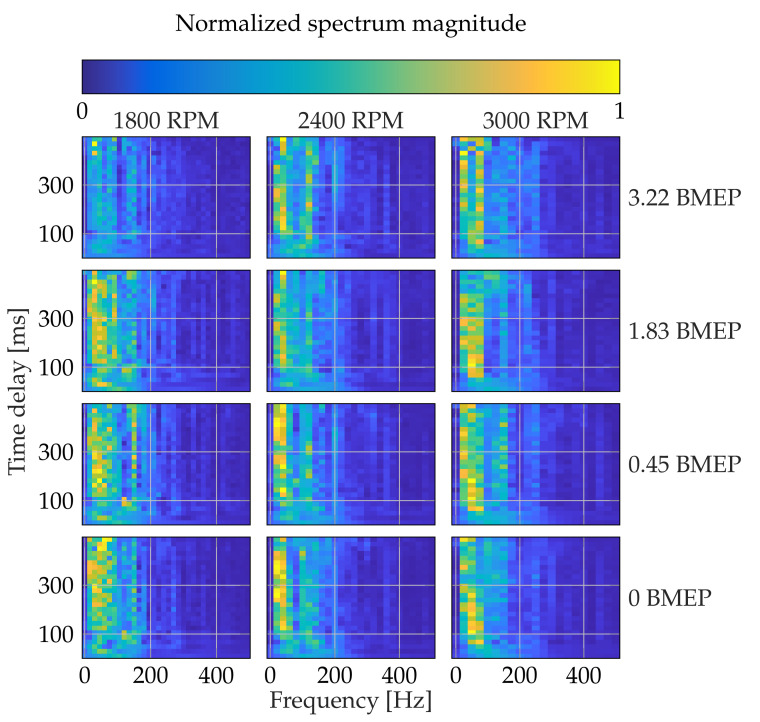
TDNN weights magnitude spectrum. Different *K* values (maximum time delay), three engine velocities (measured in RPM), and four loads (Table 4) are studied. The color indicates the normalized spectrum magnitude from blue to yellow within [0,1].

Figure 9 shows the magnitude spectrum of the TDNN’ weights by varying the *K*-value (maximum time delay), the engine velocities (measured in rpm), and the load (by changing the current in amperes applied to the dynamometer). The color indicates the normalized spectrum magnitude from blue to yellow within [0,1]. As seen, the spectrum gathers most of the energy for frequency values below 200 Hz. Regarding the speed, the TDNN amplifies more the harmonics closer to 200 Hz for 1800 and 2400 RPM than for 3000 RPM. Concerning the load parameter, the higher the load value, the more flatten is the spectrum magnitude. Specifically, for the slowest engine’s velocity, the trained TDNN tends to behave as constant gain filters. Since such a kind of filter slightly adapts the input, the network finds a higher correlation between the angular velocity and the pressure signal. Furthermore, if the load is decreased, then the harmonics gain magnitude. Thus, TDNN aims to model the emerging lower frequencies with a higher power. Therefore, our training procedure highlights the relevant harmonics demanded by the target signal. For the time delay (vertical axes in Figure 9), which accounts for the TDNN complexity, the spectra appear even across the frequency for delay windows smaller than 100 ms, proving that the TDNN misses harmonics. On the other hand, most of the networks present similar frequency responses for delay windows larger than 100 ms, although they can converge to different weighting values. Then, time delays shorter than the fundamental period hinder the learning of the whole machine dynamics (Figure 6), yielding low R2 scores and poorly fitted filters.

**Figure 10:**
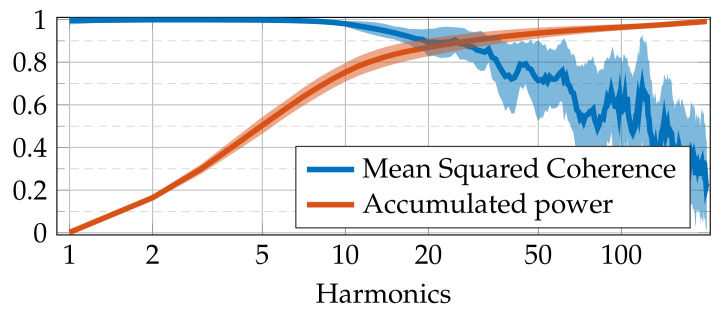
MSC results. Blue: MSC value as a function of the considered harmonic (frequency). Orange: accumulated power (normalized from [0,1]) concerning the number of harmonics. The mean and the confidence interval of the testing set are presented along with the cross-validation scheme for all the studied engine velocities and loads (time delay is fixed as 100 ms). The horizontal axis is on a logarithmic scale.

Aiming to estimate the goodness of harmonic prediction, the MSC between the target and predicted pressure signals is calculated. Figure 10 depicts the MSC and the accumulated power over the testing set for our cross-validation scheme. The mean and the confidence interval are shown for all the studied engine velocities and loads (time delay is fixed as 100 ms). Of note, the first 30 harmonics hold 90% of the signal power, where the mean MSC is over 80%. Concerning the remaining harmonics, representing 10% of the power, the average MSC decreases, and its standard deviation increases because their faster change rate makes them harder to predict. The interpretation of the above findings is two-fold. Firstly, better fitting the low harmonics, with most of the power, considerably reduces the prediction error, explaining the TDNN behavior as a low-pass filter. Secondly, underperforming at higher-ordered harmonics, usually dominated by noise, lacks relevance for the TDNN training at high signal-to-noise ratio conditions.

**Figure 11:**
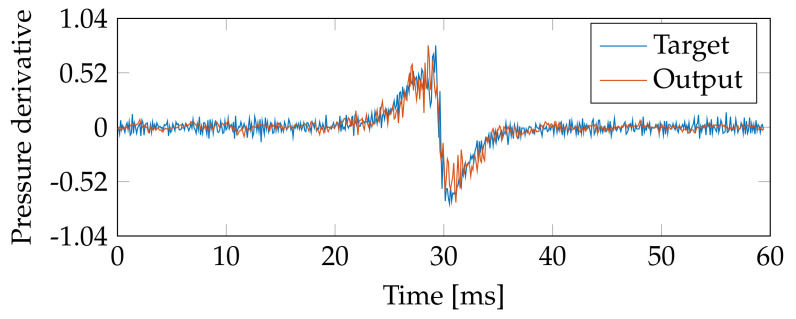
TDNN–based estimation of the pressure derivative (one record lasting 60 ms is shown as an illustrative example). Target and output pressure derivate prediction are shown for the following set-up: engine running at 1800 RPM with 0 BMEP load. The time delay is fixed as 100 ms.

**Figure 12:**
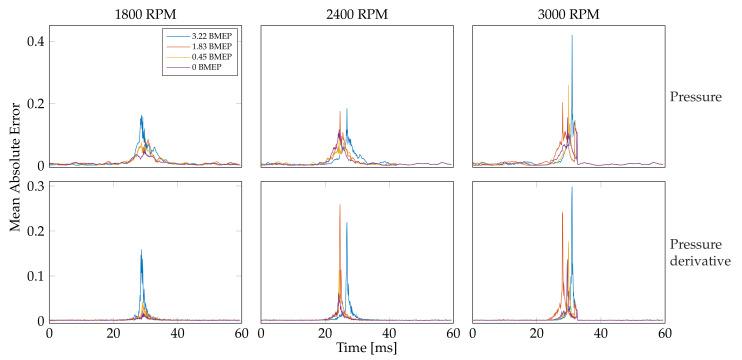
TDNN–based pressure and pressure derivative prediction results. The mean absolute error is shown for the 12 considered states by varying the engine speed and load. First row: pressure estimation results. Second row: pressure derivative estimation results. The columns stand for the studied velocities (RPM) and the line colors for the loads (BMEP).

Next, to elucidate the TDNN capability following pressure derivatives, i.e., to test the TDNN applicability for heat transfer and release rates engine analysis [20,21], the derivative of the TDNN predicted signal is computed. Figure 11 displays an illustrative example of the pressure derivative after the TDNN prediction. The target and output pressure derivative prediction are presented for one record lasting 60 ms for the engine running at 1800 RPM with 0 BMEP load (TDNN’s time delay is fixed as 100 ms). As seen, the estimated derivative follows the primary target trend. As expected, slight fluctuations are obtained, and an abrupt change is present near the pressure peak. Furthermore, Figure 12 presents the mean absolute error (MAE)-based performance along with the time instants for both the predicted pressure and pressure derivative (the 12 engine states are studied). For such a purpose, for each engine state, the pressure segments’ central peaks are detected. From the computed peak position, 60 ms are considered around it (30 ms to the left of the peak position and 30 ms to the right of the peak position) to build equal-sized datasets holding pressure and pressure derivatives segments. The MAE is computed for each time instant with respect to the considered segments. Here, the MAE is computed instead of the R2-score to avoid unstable computations when the variance for a given time instant along the studied peak-based centered segments tends to zero. The latter is significantly often near flatten regions of the pressure signal. After visual inspection of the obtained MAE performances in Figure 12, the higher the speed and the load, the higher the time-dependent error. For the highest load (3.22 BMEP), the MAE is shifted concerning the other loads, which can be explained by the abrupt change and fluctuations of the pressure derivative near the prominent signal peak. Nevertheless, considering that the introduced TDNN method is applied on a cheap and modest mechanical framework, the obtained results seem to be promising regarding the prediction of signals involving pressure derivatives.

Then, the TDNN efficiency around the pressure peaks is also computed. In particular, the normalized peak error (Pmax) is computed as the ratio (as a percentage) of the error between the reconstructed and recorded peak divided by the maximum measured cylinder pressure [28]. Besides, the peak localization error (Ploc) is computed as the difference in degrees between the location of peak pressure in the reconstructed and the measured signal. Again, as can be observed in Table 5, the increasing of the load and speed decreases the estimation performance.

Lastly, the method comparison results are shown in Table 6. The Pmax and Ploc errors are shown as the lower bound–upper bound reported (concerning the analyzed engine states). Furthermore, the best R2 is presented. As seen, the works presented in [26,27] attain suitable R2 scores. However, three input signals are studied, and only five states are tested. Besides, the peak-based measures are not computed, lacking an appropriate insight into the method’s capability to follow pressure fluctuations around the main peaks. In contrast, the work exposed by authors in [28] deals with two predictors (input signals) and studies a three-cylinder engine within nine states. Indeed, such a method obtains the best peak-based measures. Similarly, the approach presented in [29] estimates a four-cylinder engine pressure from only one predictor. Nonetheless, the achieved peak measures are the lowest. Remarkably, the introduced TDNN is the best trade-off between the required number of predictors and the obtained peak and R2-based assessments. Though only a one-cylinder engine is considered, 12 states are studied from a cheap and simple framework, involving a descent and interpretable prediction (from spectral analysis).

## 5. Conclusions and Future Work

A single-layered TDNN was trained to predict the in-cylinder pressure signal in a single-cylinder internal combustion engine using angular velocity fluctuations. The proposed approach avoids complex pre-processing stages and is only fed by the angular velocity, easily measured from the engine, reducing the signal acquisition devices’ expenses to record in-cylinder pressure directly. The approach was tested in unseen data, over 12 different engine states varying the speed and load, yielding stable prediction performances. Besides, a spectral analysis is carried out to detect the harmonics and their relevance for the prediction. Attained results found that noise is located in frequencies over 200 Hz, where the harmonics have low power content. Additionally, the tuned time-delay (network complexity represented by the number of required units) for the TDNN is slightly higher than the fundamental period that depends on the engine speed.

Moreover, the pressure derivative is computed from the predicted signal to show the TDNN applicability concerning heat transfer and release rates engine analysis [20,21]. Also, peak-based measures were computed, besides the well-known R2 score, to favor method comparison. Overall, the introduced TDNN is a cheap and straightforward approach to favor estimating the pressure signal, holding spectrum interpretability. It is worth mentioning that the proposed method can be adapted to “real-time” environments. In short, TDNN can be evaluated through the convolution between the network weights and the input velocity signal comprising a computational complexity in the order of O(NK) operations. However, optimized implementations as the well-known Fast Convolution algorithm can reduce the computational complexity to $O(N^{\prime }\log\left(N^{\prime }\right)),$ where $N^{\prime }=N+K-1$ [35].

As future work, the authors plan to test the proposal on a real-time implementation from an internal combustion engine with varying angular velocity and load (including angular velocity sensors with higher sample rates). The evaluation of the methodology under changing injection timing is also a research line of interest [8]. Additionally, the performance using different fuel injection systems as common rail direct fuel injection must be assessed [36]. The development of a spectral interpretation scheme of deeper dynamic neural networks is also required to enhance the prediction horizon. Indeed, TDNN will be extended to different multi-cylinder engines [28,29,37]. Since the introduced TDNN-based method is data-driven, it can be easily coupled with other pressure estimation tasks. More elaborate machine learning frameworks could be used from the proposed approach to favor the estimation of heat transfer and release rate engine variables [20].

## Figures and Tables

**Table 1:** CHANGFA 186F engine features.

Type	4-Stroke Vertical
Cylinders	1
Fuel	Diesel
Compression ratio	19:1
Fuel feed system	Direct injection
Fuel injection angle	20${}^{\circ }$ before TDC
Fuel pump	Positive displacement
Engine-Cooling System	Forced air
Bore × Stroke	86 mm × 70 mm
Lubricant	SAE 10W30
Max power	7.30 kW( 10.0 HP) 3600 RPM
Max torque	27 Nm 1500 RPM

**Table 2:** CFK-200 driveline features.

Application Range (GVW) (t/lb)		15–18/33,000–39,500
**Maximum braking torque**	12 V	1650 Nm/1218 lb-ft
	24 V	2000 Nm/1476 lb-ft
**Weight**	Complete	232 Kg/511 lb
	Stator	154 Kg/339 lb
	Rotors	78 Kg/172 lb
**Rotors Inertia**		2.69 Kgm${}^{2}$/64 lb-ft2
**Maximum transmissible torque**		31,600 Nm/23,321 lb-ft
**Max. admissible R.P.M.**	Constant	2600
	Periodic	3600
**Air-gap regulation (±0.1 mm/0.0039 inch.)**		1.4 mm/0.0551 inch

**Table 3:** Spark plug pressure sensor features.

Model	Optrand D822J6-SP
**Pressure Range [bar]**	0–100
**Input Voltage [V]**	9–18 DC
**Output Voltage [V]**	0.5–4.5 DC
**Sensitivity [bar/V]**	26.02
**Frequency Range [Hz]**	0.1–20,000
**Temperature Range [°C]**	0–390
**Cable Length [m]**	1.5

**Table 4:** Tested loads from an external dynamometer. The current in the dynamometer, the torque, and the equivalent load are presented.

Load level	Current [A]	Torque [Nm]	BMEP [bar]
0	0	0	0
1	0.5	1.5	0.45
2	1	6.1	1.83
3	1.5	10.7	3.22

**Table 5:** Pmax error (%) | Ploc error (°) results for the studied engine states (speed and load are varied). The mean value of the pressure signals in the testing set is reported for each studied state.

Speed [RPM]/Load [BMEP]	0	0.45	1.83	3.22
**1200**	1.158 | 2.258	−0.163|1.303	2.222|1.718	−4.991|1.165
**1500**	2.001|2.086	4.193|2.588	−0.054|2.143	2.867|1.948
**1800**	4.681|2.503	6.228|2.875	7.233|1.479	2.619|1.469

**Table 6:** Method comparison results. The Pmax error (%) and the Ploc error (°) are shown as the lower bound-upper bound performance. The best R2 is reported. The state-of-the-art abbreviations stand for: ELM: Extreme Learning Machine, ANN: Artificial Neural Networks. −: not provided.

Method	#Cylinders	#Predictors	#States	Ploc Error (°)	Pmax Error (%)	R2
ELM [26]	1	3	5	-	-	0.993
ANN [27]	1	3	5	-	-	0.998
ANN [28]	3	2	9	1.55–3.08	1.52–2.86	-
Kalman Filter [29]	4	1	9	−2.11–2.30	−17.74–12.00	-
Ours	1	1	12	1.30–2.59	−4.99–7.23	0.994

## Data Availability

Publicly available datasets were analyzed in this study. This data can be found here: https://bit.ly/3e6drME.
